# Reply to: Sex for the purposes of MELD 3.0 calculation: When this differs from birth sex and implications for policy

**DOI:** 10.1097/HC9.0000000000000960

**Published:** 2026-05-08

**Authors:** Marta Tejedor, Nazia Selzner, Marina Berenguer

**Affiliations:** 1 Department of Internal Medicine; Division of Gastroenterology and Hepatology. University of Iowa Health Care. Iowa City, USA; 2 Transplant Hepatology. Ajmera Transplant Center, University of Toronto. Toronto, Canada; 3 Hepatology and Liver Transplant Unit, Hospital Universitario y Politécnico La Fe, Valencia, Spain; 4 University of Valencia, Medicine Department, Valencia, Spain; 5 Liver and Digestive Diseases Networking Biomedical Research Centre (CIBERehd), Instituto de Salud Carlos III, Madrid, Spain; 6 Instituto de Investigación Sanitaria (IIS) La Fe, Valencia, Spain

Dear Editors,

We congratulate Duong et al. on exploring the knowledge gap in how allocation policies impact transgender and gender-diverse (TGD) populations with a goal of achieving a fair access to transplant for all candidates. Though still a minority of patients listed for liver transplant (LT), globally the TGD community is growing and is more prone to liver disease.^1^ Consequently, hepatologists should expect to encounter TGD patients with end-stage liver disease more frequently in years to come.

MELD3.0 was developed with the intent of decreasing sex disparities in accessing liver transplant. Recently, Tanaka et al. showed that implementation of MELD 3.0 was associated with a sustained increase in organ offers for women. However, the rise in completed LTs was transient, with the estimated odds of transplant for women reverting to pre-policy levels by the end of follow-up.^2^

Therefore, the impact of MELD3.0 implementation on different sub-groups, including TGD candidates, is yet to be established.

Gender Affirming Hormone Therapy (GAHT) in TGD patients leads to substantial improvements in mental health and is generally safe from a hepatology perspective when used according to guidelines. However, it modifies body composition and lean muscle mass in TGD people, leading to opposing secondary changes in creatinine values (significant increase in transgender men-by 0.15 mg/dL at one year after starting GAHT; vs decrease in transgender women -by 0.05 mg/dL, NS).^3^ These changes do not necessarily reflect true changes in renal function, and therefore its use in the MELD3.0 formula could introduce an error that affects prioritization. For instance, comparing MELD derived scores in transgender (male to female, with potentially greater muscle mass and height) with cisgender women would be interesting. It is worth exploring other allocation systems, such as GEMA, which substitutes creatinine for Royal Free GFR, a specific equation developed for cirrhotic patients.^4^ Finally, body surface area (BSA) is associated with waitlist outcomes, with the smallest candidates being disadvantaged.^5^ Therefore, BSA should be considered when assessing allocation systems in TGD populations.

As the authors mention, trying to fix one inequity could lead to another, and sex-based adjustments alone may be insufficient to address the nuances related to gender.

## References

[1] Nguyen TN Jackson WE Roth NC Cinque F Sarkar M Samala N. Chronic liver disease and hepatology care in transgender and gender diverse populations. Lancet Gastroenterol Hepatol. 2026;11:334–344. 41544636 10.1016/S2468-1253(25)00287-0

[2] Tanaka T Shane DM Lai JC. Sex and body size disparities under MELD 3.0: Evaluation of persisting gaps in liver transplant access. Hepatology. 2025. doi: 10.1097/HEP.0000000000001581. Epub ahead of print. 10.1097/HEP.0000000000001581 PMC13576647 41100798

[3] Krupka E Curtis S Ferguson T Whitlock R Askin N Millar AC. The Effect of Gender-Affirming Hormone Therapy on Measures of Kidney Function: A Systematic Review and Meta-Analysis. Clin J Am Soc Nephrol. 2022;17:1305–1315. 35973728 10.2215/CJN.01890222 PMC9625103

[4] Rodríguez-Perálvarez ML Gómez-Orellana AM Majumdar A Bailey M McCaughan GW Gow P. Development and validation of the Gender-Equity Model for Liver Allocation (GEMA) to prioritise candidates for liver transplantation: a cohort study.. Lancet Gastroenterol Hepatol. 2023;8:242–252. 36528041 10.1016/S2468-1253(22)00354-5

[5] Kling CE Biggins SW Bambha KM Feld LD Perkins JH Reyes JD. Association of Body Surface Area With Access to Deceased Donor Liver Transplant and Novel Allocation Policies. JAMA Surg. 2023;158:610–616. 36988928 10.1001/jamasurg.2023.0191 PMC10061309

